# Molecular imaging with positron emission tomography and computed tomography (PET/CT) for selecting first-line targeted treatment in metastatic breast cancer: a cost-effectiveness study

**DOI:** 10.18632/oncotarget.24869

**Published:** 2018-04-13

**Authors:** Rositsa G. Koleva-Kolarova, Marcel J.W. Greuter, Talitha L. Feenstra, Karin M. Vermeulen, Erik F.J. de Vries, David Parkin, Erik Buskens, Geertruida H. de Bock

**Affiliations:** ^1^ Department of Epidemiology, University of Groningen, University Medical Center Groningen, Groningen, The Netherlands; ^2^ School of Population Health Sciences, Faculty of Life Sciences and Medicine and Biomedical Research Center, King's College London, London, United Kingdom; ^3^ Department of Radiology, University of Groningen, University Medical Center Groningen, Groningen, The Netherlands; ^4^ National Institute for Public Health and the Environment, Center for Nutrition, Prevention and Health Services Research, Bilthoven, The Netherlands; ^5^ Department of Nuclear Medicine and Molecular Imaging, University of Groningen, University Medical Center Groningen, Groningen, The Netherlands; ^6^ Department of Economics, City University London, London, United Kingdom

**Keywords:** breast neoplasm, neoplasm metastasis, diagnostic imaging, positron emission tomography, ^89^Zr-trastuzumab

## Abstract

Our aim was to evaluate the potential cost-effectiveness of PET/CT with FES and ^89^Zr-trastuzumab compared to pathology to select first-line targeted treatment in metastatic breast cancer (MBC) patients with non-rapidly progressive disease. A previously published and validated model was extended and adapted for this analysis. Two alternative scenarios were compared. In the care as usual pathway first-line targeted treatment of MBC patients was assigned on the basis of pathology results, while in the intervention pathway treatment selection was based on the results from the PET/CT imaging. Costs, life years gained (LYG) and incremental cost-effectiveness ratios (ICER) were calculated. More MBC lesions were detected in the intervention pathway than in the care as usual pathway. The diagnostic costs to evaluate the receptor status and the treatment costs were higher in the intervention strategy, as were total costs and total LYG. The ICER for replacing biopsies with PET/CT imaging with FES and ^89^Zr-trastuzumab, assuming sensitivity of 77.1% and specificity of 80%, ranged from €71,000 to €77,000 per LYG. When assuming sensitivity of 80% and specificity of 76.7%, the ICER for replacing biopsies with PET/CT imaging with FES and ^89^Zr-trastuzumab ranged from to €74,000 to €80,000 per LYG. The application of PET/CT with FES and ^89^Zr-trastuzumab in first-line treatment selection for MBC patients has the potential to be a cost-effective intervention. Our analysis demonstrated that even a small increase in the sensitivity and the specificity of PET/CT can have a large impact on its potential cost-effectiveness.

## INTRODUCTION

Breast cancer can present in many different ways with regards to stage, phenotype, location, and heterogeneity of the disease. Many treatment options are available, and their efficacy is highly dependent on the disease characteristics. Treatment of non-rapidly progressive metastatic breast cancer (MBC) is stratified on the basis of the tumor expression of receptors that are targets for pharmacological intervention. Human epidermal growth factor receptor 2 (HER2-) and hormone receptor (HR–estrogen receptor (ER) and progesterone receptor (PR)) targeted and hormonal therapies are recommended for patients with tumors that express these receptors [1, 2]. Patients with negative receptor status (HER2- and HR-negative) of the metastatic disease do not respond to targeted therapy in general, therefore, chemotherapy remains as a treatment option [3, 4]. Targeted therapy for MBC can potentially prolong the survival of MBC patients [5], but can also be costly and may bring adverse effects. New targeted therapies regularly become available (e.g. palbociclib, pertuzumab) and a prior selection of patients is crucial to optimize outcomes of targeted therapy at acceptable costs. Moreover, this may also improve progression-free and overall survival [5], and further minimize adverse effects and avoid unnecessary harms and associated costs for those who do not respond.

Targeted treatment for MBC patients is currently selected and administered according to pathology results of tumor receptor status, often obtained from the primary tumor. However, pathology can often be inconclusive or insufficient regarding receptor expression [6], and receptor expression in the primary tumor or in a single metastatic lesion is not necessarily identical or reflects the receptor status of all metastases [7–11]. As an alternative positron emission tomography and computed tomography (PET/CT) imaging with the tracers 16α-[^18^F]fluoro-17β-estradiol (FES) and zirconium-89-(^89^Zr)-trastuzumab has been proposed to evaluate the tumor ER and HER2 expression in MBC and thus guide the selection of patients who might benefit from targeted treatment [12–15].

Since molecular imaging with PET/CT is perceived as expensive and there is still uncertainty regarding its sensitivity and specificity, prior evaluation of its potential cost-effectiveness when applied to select first-line targeted treatment in MBC patients is warranted. Previous economic evaluations of PET/CT in MBC are limited to diagnosis of the disease [16, 17]. As a result, the potential cost-effectiveness of the application of molecular PET/CT imaging to select targeted treatment in MBC patients is unclear. Therefore, the aim of this study was to evaluate the potential cost-effectiveness of PET/CT with FES and ^89^Zr-trastuzumab tracers compared to pathology testing to select first-line targeted treatment in MBC patients with non-rapidly progressive disease. We worked on a model including a “biopsy versus imaging” strategy for receptor status evaluation because we wanted to test the potential cost-effectiveness of PET/CT with FES and ^89^Zr-trastuzumab as a one-stop shop for treatment selection in MBC. We limited our analysis to a few important treatment options to demonstrate proof of concept of potential cost-effectiveness of imaging, instead of working on a model including all new targeted therapies.

## RESULTS

For baseline scenario 1 we assumed that the sensitivity of FES-PET/CT and ^89^Zr-trastuzumab PET/CT was set at 77.1% and the specificity was set at 80%, as this was the minimum at which PET/CT could demonstrate potential cost-effectiveness. For baseline scenario 2 we assumed that the sensitivity of FES-PET/CT and ^89^Zr-trastuzumab PET/CT was set at 80% and the specificity was set at 76.7%, as this was the minimum at which PET/CT could demonstrate potential cost-effectiveness. The results from the decision analytical model are presented in Table 1.

**Table 1:** Simulation results for the care as usual and the intervention pathways for a hypothetical cohort of 1,000 women

	Care as usual	Intervention	Care as usual	Intervention
	Baseline scenario 1		Baseline scenario 2	
Diagnosed MBCs				
Bone	228	234	228	235
Lung	199	203	199	204
Brain	65	66	65	66
Liver	159	163	159	163
Cost of receptor status evaluation (in k€)	695	3,500	695	3,500
Cost of therapies (in k€)				
Chemotherapy	1,700	1,700	1,700	1,800
Trastuzumab monotherapy	360	370	360	366
Trastuzumab with paclitaxel or vinorelbine)	1,700	1,740	1,700	1,740
Anastrozole	790	815	790	811
Letrozole	990	1,020	990	1,014
Trastuzumab + hormonal therapy	4,300	4,500	4,300	4,170
Total costs (in k€)	7,800^*^/9,400^**^	10,900^*^/12,500^**^	7,800^*^/9,400^**^	10,700^*^/12,200^**^
Total LYG	1,500^*^/1,700^**^	1,600^*^/1,700^**^	1,500^*^/1,700^**^	1,600^*^/1,700^**^
ICER	n/a	71,000^*^/77,000^**^	n/a	74,000^*^/80,000^**^

^*^If ER+/HER2– patients were treated with Anastrozole and ER-/HER2+ patients were treated with Trastuzumab monotherapy;

^**^If ER+/HER2- patients were treated with Letrozole and ER-/HER2+ patients were treated with Trastuzumab combination therapy (trastuzumab and paclitaxel).

n/a – not applicable.

### Health effects and costs

More MBC lesions were detected in the intervention pathway than in the care as usual pathway. The diagnostic costs to evaluate the receptor status and the treatment costs were also higher in the intervention strategy, as were total costs and total life years gained (LYG). The treatment with anastrozole and trastuzumab monotherapy was cheaper than the use of letrozole and trastuzumab combination therapy (trastuzumab and paclitaxel) (Table 1).

### Cost-effectiveness

The incremental cost-effectiveness ratio (ICER) for replacing biopsies with PET/CT imaging with FES and ^89^Zr-trastuzumab for receptor status evaluation and treatment selection in MBC patients (assuming sensitivity of 77.1% and specificity of 80%) ranged from to €71,000 to €77,000 per LYG if patients were treated with letrozole and trastuzumab combination therapy (trastuzumab and paclitaxel), or with anastrozole and trastuzumab monotherapy, respectively (baseline scenario 1, Table 1).

The ICER for replacing biopsies with PET/CT imaging with FES and ^89^Zr-trastuzumab for receptor status evaluation and treatment selection in MBC patients (assuming sensitivity of 80% and specificity of 76.7%) ranged from to €74,000 to €80,000 per LYG if patients were treated with letrozole and trastuzumab combination therapy (trastuzumab and paclitaxel), or with anastrozole and trastuzumab monotherapy, respectively (baseline scenario 2, Table 1).

### Sensitivity analyses

When the probabilities of inconclusive and unobtainable biopsies were set at their minimum values (i.e. 10%), the minimum sensitivity and specificity of FES-PET/CT and ^89^Zr-trastuzumab PET/CT, at which the intervention strategy was likely to be cost-effective were 90% and 90.6%, respectively. For values of PET/CT sensitivity and specificity below 90% and 90.6%, respectively, the intervention strategy was dominated, because it was costlier and less effective than the care as usual. For even lower values of PET/CT sensitivity and specificity, below 70% and 80%, respectively, effectiveness was logically even less. This lack of effectiveness resulted in cost savings from avoided treatment costs as compared to care as usual (Supplementary Appendix A, Supplementary Tables A1 and A2).

When the probabilities of inconclusive and unobtainable biopsies were set at 15%, the minimum sensitivity and specificity of FES-PET/CT and ^89^Zr-trastuzumab PET/CT, at which the intervention strategy was likely to be cost-effective were 83% and 86%, respectively. Below these values for PET/CT sensitivity and specificity the intervention strategy was less effective than the care as usual (Supplementary Appendix A, Supplementary Tables A1 and A2).

When the probabilities of inconclusive and unobtainable biopsies were set at 20%, for values of PET/CT sensitivity and specificity below 77% and 80%, respectively, the intervention strategy was less effective than the care as usual (Supplementary Appendix A, Supplementary Tables A1 and A2).

When the probabilities of inconclusive and unobtainable biopsies were set at their maximum values (i.e. 30%), the minimum sensitivity, at which the intervention strategy was likely to be cost-effective was 59.6% and the corresponding specificity was 70%. Alternatively, the lowest specificity value for which the intervention strategy was still cost-effective than care as usual was 58.5 %, at a sensitivity of 70%. For values of PET/CT sensitivity and specificity below these, the intervention strategy was less effective than the care as usual (Supplementary Appendix A, Supplementary Tables A1 and A2).

## DISCUSSION

The aim of this study was to assess the potential cost-effectiveness of applying PET/CT with FES and ^89^Zr-trastuzumab as compared to pathology testing to select first-line treatment in MBC patients with non-rapidly progressive disease. A previously published and validated computer model on metastatic breast cancer simulation was extended and applied in this analysis. The intervention pathway yielded higher costs to evaluate receptor status of the MBC disease and select treatment as compared to the care as usual pathway. The number of LYG was also higher in the intervention pathway, provided sensitivity and specificity were sufficiently high. The application of PET/CT with FES and ^89^Zr-trastuzumab tracers for selecting the first-line therapy in MBC had the potential to be cost-effective when the sensitivity and the specificity of the PET/CT were at least 77.1% and 80% (aiming at the lowest sensitivity), or – starting with low values of specificity: 80% and 76.7%, respectively. The results of this analysis reflected the expected LYG, costs and cost-effectiveness in MBC patients based on the receptor status of their disease (either ER or HER2, or both).

The clinical value of PET/CT has thus far only been established with the FDG tracer for detection, staging, re-staging and therapy monitoring in various neoplastic processes, including breast cancer [18–21]. FDG-PET was included in some guidelines for breast cancer work-up, but the value of FDG-PET in metastatic breast cancer has been debated. FDG-PET could aid the staging of the disease (i.e. detect lesions), but it does not provide information about the status of the drug targets in the lesions [18–21]. PET/CT with the FES and the ^89^Zr-trastuzumab tracers could provide a whole-body visualization of metastatic recurrence after breast cancer and its ER- and HER2-status. Therefore, it has implications for therapy selection, personalized treatment and follow-up which may result in increase of survival [12–15, 22]. Furthermore, PET/CT with FES and ^89^Zr-trastuzumab could be a valuable alternative *in situations* where biopsy is not feasible. Biopsy could only give information about several drug targets in a small part of a single lesion. In contrast, PET/CT imaging could give information about a few targets in all lesions in the body. It is known that discrepancy in target expression between lesions in a single patient is observed in a high percentage of the patients (10 – 30%) [8–12].

We varied the sensitivity and the specificity of PET/CT with FES and ^89^Zr-trastuzumab from 0 to 100% to evaluate different combinations of these at which the intervention pathway was likely to be cost-effective. The values for sensitivity and specificity in our analysis are theoretical and suggest a precision which is not reached in daily practice. Previous studies showed a high sensitivity of FES-PET (overall 84%), but with considerable variability between studies (range 69–100 %) [14]. This variability was probably due to differences in inclusion criteria and the location of lesions. FES-PET/CT demonstrated high sensitivity in patients with bone and lung lesions, and relatively poor sensitivity in patients with liver and brain lesions. The specificity of FES-PET/CT in ER-positive lesions is generally very high in all studies (overall 98%) [14]. For ^89^Zr-trastuzumab PET, no sensitivity and specificity data are available yet. Our analysis demonstrated that even small increase in the sensitivity and the specificity of PET/CT could have a large impact on its potential cost-effectiveness. Whether the impact of FES-PET/CT and ^89^Zr-trastuzumab-PET/CT on treatment selection in MBC would actually result in prolonged progression-free and overall survival and maintained quality of life is yet to be determined [23].

There are currently few economic evaluations of PET/CT which are mainly aimed at assessing its cost-effectiveness in diagnosing and staging of different types of cancers [16, 17, 19–21]. PET/CT with FDG tracer was found to be cost-effective in pre-operative staging of colorectal cancer, non-small cell lung cancer and solitary pulmonary nodes [19–21]. FDG-PET/CT was also found beneficial in evaluating the treatment response in the follow-up of non-small cell lung cancer [20, 21]. Previously, we have demonstrated that FES-PET/CT can diminish the number of both false positive and false negative results when applied as a first choice diagnostic to ER-positive MBC patients. In addition, it could decrease the number of imaging and pathology tests required to diagnose distant relapse [16]. Other studies have demonstrated that PET/CT could generate more quality-adjusted life years (QALYs) in comparison to the standard work-up for diagnosing MBC at an incremental cost of ₤29,700 (∼ €36,000) per QALY [17]. The limited economic evaluations of PET/CT resulted from lack of clinical data from randomized controlled trials (RCTs) or observational studies which explore the potential of PET/CT for treatment selection and response monitoring.

This study evaluated the potential cost-effectiveness of the application of molecular imaging with PET/CT for selecting the first-line treatment in MBC. Although our findings clearly demonstrated the potential of PET/CT with FES- and ^89^Zr-trastuzumab tracers to be cost-effective when selecting the first-line treatment in MBC patients we must be aware of the limitations of this analysis.

We simplified our approach and limited it to a “biopsy versus imaging” for receptor status evaluation and a few important treatment options to demonstrate proof of concept. It should be noted here, that combinations of biopsy and imaging, and other treatment options can be substituted into the model, if necessary. This study is a starting point for further research on more specific situations using a more sophisticated model.

The data regarding the inconclusive and unobtainable rate of biopsies to detect MBC and reveal receptor status were obtained from literature and not from a dedicated RCT or cohort study aimed at evaluating these parameters.

The possible treatment combinations were limited to those listed in NICE guidance documents, irrespective of the recommendation status, and therefore did not necessarily reflect therapeutic advances and variety in management of MBC patients. Costs of therapies were obtained from the NICE guidelines, converted to euros according to the 2016 exchange rate and corrected for the difference in per capita expenses between the United Kingdom and the Netherlands using World data bank estimates, that is costs of all therapies were valued in 2016 euros and used a fixed PPP (Purchasing Power Parity) conversion rate, not a fluctuating exchange rate. Thus, they were normalized for inflation and exchange rate fluctuations.

Another limitation of the current analysis is that the data on survival periods were obtained from reviews and meta-analyses of RCTs examining the progression-free and overall survival after a specified type of treatment. These survival periods did not necessarily reflect the survival of MBC patients according to the location of the distant spread and the breast cancer subtype (luminal A, luminal B, luminal/ HER2, HER2-enriched, basal-like, and triple negative nonbasal). As it is known that every cancer subtype demonstrates a distinct pattern of metastatic relapse and the location of the metastatic spread is associated with notable differences in survival, including these probabilities in the model alongside the probability of different survival according to the type of treatment would allow for a more precise estimation of the expected incremental cost-effectiveness. This would have implications not only for the improved survival rate but for costs as well as MBC patients who survive longer would incur additional substantial costs for their end of life care and treatment.

Furthermore, we assumed a single number for the sensitivity and the specificity of PET/CT for both FES and ^89^Zr-trastuzumab, but it is known that sensitivity and specificity are highly dependent on, amongst others, the expression levels of the target, the instrumentation and the characteristics of the tracer used. Another limitation was that a significant fraction of the patients has a heterogeneous receptor status between lesions (i.e. some lesions are positive, while others are negative). Pathology cannot provide information about this heterogeneity. On the other hand, little is known about the impact of heterogeneity on treatment selection.

We did not take into consideration the complications resulting from obtaining invasive pathology tests, which could also affect the costs and the quality of life of MBC patients.

Non-responsiveness to selected therapy and adverse effects resulting from assigned therapies were also not evaluated and these could affect costs and survival, and thus cost-effectiveness estimates.

We only considered first-line treatment selection in our analysis, therefore, second-line therapies and the potential application of molecular imaging for treatment selection in second-line and beyond metastatic setting was outside the scope of this study.

The uncertainty and the limitations in our study were mainly due to the lack of clinical data from RCTs and observational studies investigating the role of PET/CT for treatment selection in MBC patients. Initial data from other studies regarding the sensitivity and the specificity of Zr-trastuzumab PET demonstrated multiple false positives in presumed HER2-negative patients but the sample size was small [24]. Part or all of these uncertainties and limitations in data availability could be solved when a recent RCT aimed at patient-tailored cancer treatment supported by molecular imaging is concluded [23]. This would allow to explore other premises in which PET/CT would not replace pathology in all patients but would be added in specific cases.

The application of PET/CT with FES and ^89^Zr-trastuzumab as a replacement for care as usual in first-line treatment selection in MBC patients comes at additional costs but has the potential to be a cost-effective intervention. The minimum sensitivity and specificity at which PET/CT imaging with FES and ^89^Zr-trastuzumab tracers would be cost-effective were estimated at 77.1% and 80%, or 80% and 76.7%, respectively. PET/CT imaging with FES and ^89^Zr-trastuzumab tracers could be a promising advancement in the treatment selection in MBC [22–24]. Our analysis, however, demonstrated that even a small increase in the sensitivity and the specificity of PET/CT can have a large impact on its potential cost-effectiveness. As the current analysis has uncertainties and limitations resulting from the lack of RCTs’ or cohort study clinical data, additional prospective research (in terms of more and larger clinical studies) is required to validate the role of PET/CT in treatment selection and assess the generalizability of the reported benefits, particularly given the associated costs of the modality.

## MATERIALS AND METHODS

A published and validated computer model on metastatic breast cancer simulation (MBCSIM) served as a basis for this analysis [16]. The MBCSIM model was designed to simulate the follow-up of women after initial breast cancer and diagnose MBC in women who exhibited symptoms suggestive of distant relapse from the Dutch health care perspective. Detailed description of the MBCSIM model and its parameters could be found in a previous publication [16].

### Structure of the model

**Figure 1:**
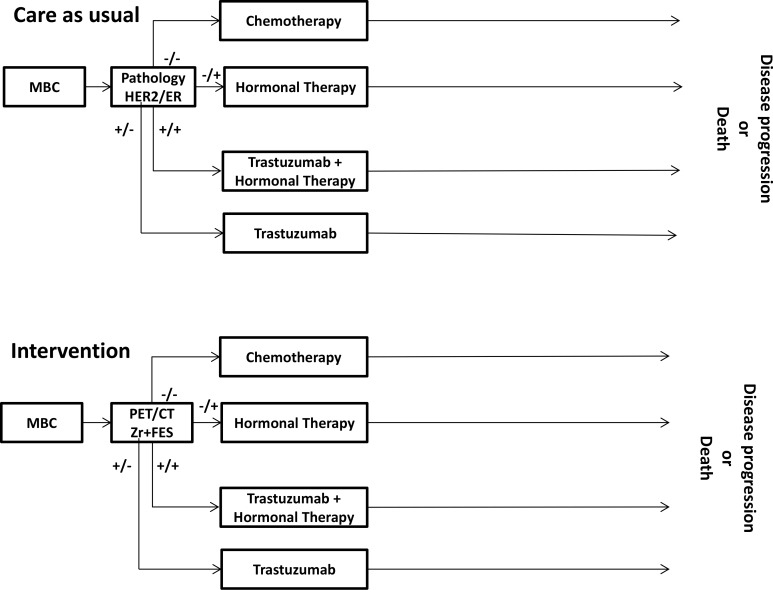
Flow chart of the decision analytical model

For this application, we designed a decision tree which extended the MBCSIM model by incorporating targeted treatment selection components after diagnosing the distant relapse. The structure of the current model and its parameters are presented in Figure 1 and Table 2. The presence of distant relapse was based on the probability of developing MBC and distributed over four locations (bone, lung, liver, brain). When MBC was diagnosed, the patient followed either a care as usual pathway or an intervention pathway involving targeted therapy.

**Table 2:** Parameters of the decision analytical model

	Baseline estimate (%)	Minimum estimate (%)	Maximum estimate (%)	Reference
Distribution of location of MBC				
Bone	46	25	61	25
Lung	40	26	60	
Brain	13	7	28	
Liver	32	24	49	
Probability of MBC being ER+	62	45	70	7
Probability of MBC being ER–	26	23	30	7
Probability of MBC being HER2+	18	14	20	8
Probability of MBC being HER2–	76	72	80	8
Probability of inconclusive pathology	20	10	30	16
Probability of unobtainable pathology	20	10	30	9–11
Survival periods (in months)				
after chemotherapy	22.7	20.3	31.9	5, 26–30
after trastuzumab therapy	32.2	25.4	40.8	
after hormonal therapy	28.6^*^/33.2^**^	17.4^*^/32.3^**^	39.2^*^/34^**^	
after trastuzumab and hormonal therapy	34.1	n/a	n/a	

^*^anastrozole, ^**^letrozole, n/a – not available.

### Simulated patient population

We used two hypothetical cohorts of 1000 women each to perform the simulations for the care as usual and the intervention pathways. The women in each cohort were diagnosed and treated for primary breast cancer. Within the 5-year period after primary diagnosis these women developed distant relapse. The presence of metastatic disease was simulated on the basis of the risk to develop distant relapse over time and distributed over the four most common locations, bone, lung, liver, and brain. The receptor status of the primary tumor as well as the probability of receptor change in the metastatic disease was based on literature. In the simulation, each cohort was assigned to either the care as usual or the intervention pathway irrespective of the receptor status of the primary breast cancer.

### Strategies for treatment selection in MBC

Two pathways for selecting targeted treatment in MBC patients were compared.

### Care as usual pathway

In the care as usual pathway after being diagnosed with MBC, patients were assumed to undergo pathology testing to determine the ER- and HER2-expression of the biopsied lesions. Then patients were assigned to therapy based on the receptor status of their disease as determined by the pathology results. Patients with metastatic lesions which expressed positive ER and positive HER2 status received first-line trastuzumab and hormonal therapy (anastrozole). Patients with metastatic lesions which expressed positive ER and negative HER2-status were assigned to first-line hormonal therapy alone (either anastrozole or letrozole). Patients with metastatic lesions who tested negative on the ER and positive on the HER2-status received first-line trastuzumab (mono or combination (trastuzumab and paclitaxel) therapy). Patients with metastatic lesions which had negative ER and HER2-expression received first-line chemotherapy.

### Intervention pathway

In the intervention pathway two PET/CT scans with different tracers, FES and ^89^Zr-trastuzumab, replaced pathology testing to establish receptor status of the MBC disease, and the relevant therapy was assigned on the basis of the PET imaging results.

### Parameters of the decision analytical model

The parameters of the model and their baseline, minimum and maximum estimates were derived from literature (Table 2). The distribution of the locations of MBC was obtained from a cohort of patients diagnosed with primary disease and subsequently developed MBC within a 5-year follow-up [25]. The overall survival estimates were based on meta-analyses and RCTs of receptor positive MBC patients treated with targeted, hormonal and chemo- therapies [5, 26–30]. The inconclusive rate of pathology and the rate of unobtainable pathology samples were based on previous publications [9–11, 16]. As studies reporting on the sensitivity and specificity of PET/CT with different tracers were either limited [14, 15, 24] or still in progress [23], we chose to vary these parameters independently of age and of the location of the MBC to analyze cost-effectiveness.

### Outcomes

The outcomes evaluated were life years gained (LYG), costs (diagnosis and treatment related), and cost-effectiveness (incremental cost-effectiveness ratio – ICER).

### Health effects

Life years gained were modeled by assigning survival periods to the different health states. These survival periods were obtained from meta-analyses and RCTs which estimated overall survival of MBC patients treated with targeted, hormonal and chemo- therapies [5, 26–30].

### Costs

Only direct costs of imaging tests and treatment regimens were included, valued in euros. The costs of the imaging and pathology tests were obtained from the tariffs level for 2016 (Table 3) [31]. Only Food and Drug Agency and European Medicines Agency approved hormonal, targeted and chemotherapies were considered for costing. The costs of the approved therapies were obtained from the National Institute for Health and Care Excellence (NICE) guidance reports [32–38], and corrected for the difference in per capita expenses between the United Kingdom and the Netherlands using World data bank estimates from 2015 [39].

**Table 3:** Costs of imaging and pathology tests, and treatment

	Cost in €	Reference
**Imaging test**		
PET whole body	1,144	31
CT bone	175	31
CT lung	194	31
CT brain	145	31
CT liver	207	31
**Pathology test**		
Liver	137	31
Bone/lung/brain	657	31
**Treatment options**		
Trastuzumab monotherapy	9,730	33, 34, 37–39
Trastuzumab combination therapy (trastuzumab and paclitaxel)	46,080	
Trastuzumab + anastrozole	48,512	33, 34, 36–39
Docetaxel	11,015	35, 37–39
Anastrozole	2,141	36, 37–39
Letrozole	2,676	36, 37–39

The treatment costs represent cost per patients for the whole duration of treatment.

Table 3 presents the costs per treatment for the whole duration of treatment and the average duration of treatment is provided in Appendix B. Detailed description of costs and cost calculations is available in Supplementary Appendix B. Costs of pathology complications, treatment of adverse events or palliative care, and second and third line treatment were not taken into account. Discounting was not applied due to the relatively short-time horizon (less than 5 years) of the analysis.

### Cost-effectiveness analyses

A health payer perspective was adopted. The total costs of each pathway was calculated by summing the costs of all performed tests and the selected treatments. The ICER was calculated by dividing the incremental cost by the number of incremental LYG. Potential cost-effectiveness was established by varying the sensitivity and the specificity of the PET/CT and assessing the impact of these variations on the ICERs.

In the Netherlands, an informal ceiling ratio of € 80,000 per LYG was established by the Dutch Council for Public Health and Health Care [40]. This is considered a maximum ceiling ratio applicable when there is a high burden of disease, as is the case of MBC.

### Sensitivity analyses

A univariate sensitivity analysis was performed for practical reasons. Since the model has demonstrated to be predominantly sensitive to the input parameters related to the sensitivity and the specificity of the PET/CT and the pathology failure, it was considered that a probabilistic sensitivity analysis where all the input parameters were changed at the same time would not provide essentially different information. Therefore, univariate sensitivity analyses with minimum and maximum values of the 95% CI for the input parameters listed in Table 2 for the entire cohort of 1,000 women were performed to evaluate the effects of parameter uncertainty in each pathway.

Since sensitivity and specificity show a certain trade-off, we evaluated different combinations of these, setting in turn sensitivity or specificity at a pre-specified value and then varying the remaining parameter. We analyzed the values at which the intervention was just cost-effective (at €80,000 per LYG), effective (had positive LYG) and some fixed values below and above these values, to gain insight into the changes in incremental cost-effectiveness of the intervention as compared to the care as usual, for three different values of biopsy failure probability. We used values of 77.4% for sensitivity and 80% for specificity in the baseline scenario 1 and 80% and 76.7%, respectively, for baseline 2 to calculate the point estimate for the cost-effectiveness. The sensitivity of FES-PET/CT ranges between 69% and 100% and the specificity varies between 80% and 100% [14]. For the sensitivity analyses we applied a fixed sensitivity and varying specificity of PET/CT (Supplementary Table A1) as well as a fixed specificity and varying sensitivity (Supplementary Table A2) to estimate the sensitivity of the ICER resulting from the error margins in the PET/CT sensitivity and specificity.

## SUPPLEMENTARY MATERIALS FIGURES AND TABLES


