# Supplementary material for: Molecular imaging with positron emission tomography and computed tomography (PET/CT) for selecting first-line targeted treatment in metastatic breast cancer: a cost-effectiveness study
Source: Oncotarget. 2018 Apr 13;9(28):19836–46. doi: 10.18632/oncotarget.24869 (PMC5929430; doi:10.18632/oncotarget.24869)
Supplement: Supplementary file 1 [file oncotarget-09-19836-s001.pdf]

Molecular imaging with positron emission tomography and computed tomography (PET/CT) for selecting first-line targeted treatment in metastatic breast cancer: a cost-effectiveness study

SUPPLEMENTARY MATERIALS

Appendix A. Sensitivity analyses

Table A1: Sensitivity analyses–fixed sensitivity and varying specificity of PET/CT

Sensitivity of PET/CT	Specificity of PET/CT	Costs incremental (in thousand €)	LYG incremental	ICER (in thousand €)
Probability of unobtainable and inconclusive pathology 10%				
90%	89%	2,763/2,766	2/2	1,390/1,370
90%	90.6%	3,000/3,030	38/41	80/74
95%	90%	3,300/3,400	140/160	23/22
99%	95%	4,300/4,700	360/390	11,9/11,8
Probability of unobtainable and inconclusive pathology 15%				
85%	82.4%	2,700/2,700	2/2	1,600/1,700
86%	83%	2,850/2,890	36/39	79/74
90%	80%	2,700/2,800	61/65	45/43
95%	85%	3,700/4,000	280/310	13,2/13
95%	90%	4,400/4,800	400/440	11,2/11,1
99%	95%	5,500/6,100	620/670	9/9,1
Probability of unobtainable and inconclusive pathology 20%				
80%	74.9%	2,625/2,623	1/0	3,300/6,400
80%	76.7%	2,850/2,880	36/39	80/74
85%	80%	3,600/3,800	210/230	17,3/16,8
90%	85%	4,600/5,000	420/460	10,8/10,8
95%	85%	4,900/5,400	540/590	9,1/9,3
95%	90%	5,600/6,300	650/710	8,6/8,8
99%	95%	6,700/7,500	870/950	7,7/7,9
Probability of unobtainable and inconclusive pathology 30%				
70%	56.5%	2,470/2,450	2/1	1,300/4,000
70%	58.5%	2,650/2,680	34/36	77/74
75%	65%	3,600/3,800	230/250	15,5/15,1
80%	70%	4,400/4,800	420/450	10,6/10,6
90%	80%	6,300/7,100	830/900	7,6/7,9
99%	90%	8,300/9,500	1,300/1,400	6,6/6,9

The first value in the rows incremental costs and LYG, and ICER indicates a scenario in which patients with ER+/HER2– receptor status were treated with Anastrozole and ER–/HER2+ patients were treated with Trastuzumab monotherapy, while the second value indicates a scenario in which ER+/HER2– patients were treated with Letrozole and ER–/HER2+ patients were treated with Trastuzumab combination therapy (trastuzumab and paclitaxel).

Table A2: Sensitivity analyses–fixed specificity and varying sensitivity of PET/CT

Sensitivity of PET/CT	Specificity of PET/CT	Costs incremental (in thousand €)	LYG incremental	ICER (in thousand €)
Probability of unobtainable and inconclusive pathology 10%				
89%	90%	2,839/2,841	1/1	4,100/4,300
90.6%	90%	2,950/2,990	38/42	77/71
90%	95%	3,700/3,800	140/150	27/25
95%	99%	4,600/5,000	360/390	13,1/12,9
Probability of unobtainable and inconclusive pathology 15%				
82.5%	85%	2,900/2,900	1/1	4,600/4,000
83.1%	86.2%	3,100/3,140	39/42	80/74
80%	90%	3,400/3,500	48/52	72/67
85%	95%	4,500/4,800	270/300	17/16
90%	95%	4,800/5,200	390/430	12,4/12,2
95%	99%	55,800/6,400	610/670	9,6/9,7
Probability of unobtainable and inconclusive pathology 20%				
75.1%	80%	2,977/2,981	1/1	2,800/2,400
77.1%	80%	3,090/3,130	40/44	77/71
80%	85%	3,900/4,100	200/220	20/19
85%	90%	4,900/5,400	420/460	11,9/11,8
90%	95%	6,000/6,700	650/710	9,3/9,4
95%	99%	7,000/7,900	860/950	8,1/8,3
Probability of unobtainable and inconclusive pathology 30%				
57.2%	70%	3,220/3,221	1/1	2,900/3,100
59.6%	70%	3,300/3,400	42/46	79/73
65%	75%	4,200/4,400	220/240	19/18
70%	80%	5,100/5,500	410/440	13/12
80%	90%	7,000/7,800	810/890	8,6/8,8
90%	99%	9,000/10,000	1,200/1,400	7,2/7,5

The first value in the rows incremental costs and LYG, and ICER indicates a scenario in which patients with ER+/HER2– receptor status were treated with Anastrozole and ER–/HER2+ patients were treated with Trastuzumab monotherapy, while the second value indicates a scenario in which ER+/HER2– patients were treated with Letrozole and ER–/HER2+ patients were treated with Trastuzumab combination therapy (trastuzumab and paclitaxel)

Appendix B. Cost estimations

We found the costs per tablet or milligram of the relevant therapies in the Netherlands (ZIN, 2017), and estimated the costs per package and vial. We obtained the prices for the same therapies in the UK from the National Formulary [32–38]. Then, we compared the Dutch prices [31, 40, 41] to the prices of the same therapies in the United Kingdom [32–38]. Table A shows the differences in prices for the used therapies between the Netherlands and the UK. Apparently, prices per package/vial were lower in the Netherlands for some therapies and higher for trastuzumab and docetaxel, however, the World Data Bank [39] have reported that treatment expenses amounted to €5,169 in the Netherlands versus €3,572 in the UK [39]. Therefore, in order not to underestimate costs-effectiveness evaluations we chose to use the UK prices, converted to euros according to the 2016 exchange rate and corrected for the difference in per capita expenses between the United Kingdom and the Netherlands using World data bank estimates from 2015 [39].

The cost of the trastuzumab monotherapy included the course of treatment with trastuzumab administered over a period of 12 weeks. The cost of trastuzumab combination therapy included the costs of the two therapies, anti-HER2-agent trastuzumab and chemotherapy with paclitaxel, administered over a period of 38 weeks. The cost of combination therapy with trastuzumab and anastrozole included the costs of the two treatments for a patient with an average weight of 67 kg, administered over a mean treatment period of 15 months and excluding administration, monitoring and wastage costs. Thus, the acquisition cost for a lifetime of treatment with trastuzumab plus anastrozole was estimated as the mean cost of a weekly and a 3-weekly schedule of treatment. The cost of treatment with the chemotherapy agent docetaxel was based on the cost per patient for six cycles of treatment. The acquisition cost of anastrozole was based on the cost per package of 28 tablets/1-mg each administered over a period of 15 months. The acquisition cost of letrozole was based on the cost per package of 28 tablets/2.5-mg administered over a period of 15 months. All costs of different treatments were estimated per patient.

Table B

	NL			UK
	**Per tablet**	**Per package**	**Pharmacy charges**	**Per package**
Anastrozole	€0.52	€14.56	€6-12	£68.56 (~€79.67)
Letrozole	€0.70	€19.6	€6-12	£83.16 (~€96.63)
Trastuzumab	€4.29 per 1 mg	€643.50 per 150 mg vial	n/a	£407 (~€481.79)
Docetaxel	€5.04 per 1 mg	€201.6 per 40 mg/ml vial	n/a	£162.75 (~€189.12) for a 0.5 ml vial<br>£534.75 (~€621.38) for a 2 ml vial
